# Barriers to and Suggestions on Improving Utilization of Eye Care in High-Risk Individuals: Focus Group Results

**DOI:** 10.1155/2014/527831

**Published:** 2014-10-15

**Authors:** Angela R. Elam, Paul P. Lee

**Affiliations:** ^1^Duke University Eye Center, Duke University School of Medicine, Durham, NC 27710, USA; ^2^Department of Ophthalmology and Visual Sciences, University of Michigan Kellogg Eye Center, Ann Arbor, MI 48105, USA

## Abstract

*Purpose*. To understand barriers facing high-risk individuals and to solicit the suggestions of these individuals, especially nonusers, on how to change the eye care delivery system to better meet their needs. *Methods*. Four focus groups were conducted. All discussion was audiotaped and transcribed. Content analysis was performed by the authors and with the assistance of qualitative software, NUD^*^IST Vivo. *Results*. The most frequently cited barriers include (1) cost, (2) trust, (3) communication, (4) clinic accessibility (transportation/distance), and (5) doctor-patient relationship. In underutilizers, trust was the most identified barrier to care. Suggestions on increasing educational opportunities/awareness of eye care and addressing cost and insurance issues as a means of improving trust and communications were most frequently offered, including using the Department of Social Services as a focal point for eye care education and assessment. *Discussion*. Trust is a major barrier to eye care, especially among underutilizers of disadvantaged populations. Increasing trust and eye care education at the community and individual levels is essential to increasing eye care utilization.

## 1. Introduction

A concerted effort has been made in recent years to improve vision health in the United States. The Centers for Disease Control and Prevention (CDC) have developed the Vision Health Initiative (VHI), a coordinated national public health framework to prevent vision impairment and blindness [1]. A key aspect of the CDC's initiative involves addressing disparities in eye care to improve national eye health [1]. Directing more attention to disparities in high-risk populations is an integral part of this effort [1–3]. High-risk factors for eye disease and/or vision loss that have consistently been identified include (1) increasing age, (2) racial/ethnic minority, (3) presence of diabetes mellitus, and (4) low socioeconomic status [3]. Furthermore, each of these factors has also been associated with decreased access to and underutilization of eye care services [3]. Lack of health insurance and residence in a rural area are other critical variables related to underutilization of eye care [1].

As we seek to increase eye care utilization in high-risk populations, it is important first to understand some of the barriers to care that they face. Individuals cite trust, communication, and cost/lack of insurance as major barriers to accessing eye care [4, 5]. Transportation is also documented as a major barrier to eye care [4, 6]. In addition to cost, access to timely care and overshadowing of eye disease by systemic diabetes burden were commonly noted as barriers in diabetic patients [7]. Other barriers include no perceived need and fear of pain from examination [5, 8].

Researchers and clinicians have offered suggestions on ways to overcome these barriers to care and increase appropriate use of eye care. However, few studies have sought the suggestions of high-risk individuals on how to change the eye care delivery system to better meet their needs. As such, we report on the initial findings for a study designed to emphasize this information, using focus groups to identify barriers to eye care in individuals with high risk for vision loss/eye disease and underutilization of care and their ideas on how the eye care system can be changed to better meet their needs.

## 2. Methods

Prior to the start of this study, approval of the study was granted by the Duke University Health System institutional review board. We first conducted a MEDLINE search surveying literature published in English from 1966 to 2007, using combinations of key words relevant to eye care utilization and barriers. After reviewing relevant articles the most common barriers to receiving eye care indicated were cost, transportation, insurance coverage, provider accessibility, education, communication, and trust. We used these areas and other information gathered from the literature search to develop the script used for the individual interviews and focus groups. This script was pilot tested on 3 individuals to ensure the posed questions were clear and relevant. To ensure comparability between each group, the same script was used to facilitate each focus group discussion.

We worked with local community leaders to identify areas for study participant recruitment, specifically areas with a high proportion of individuals at high risk for vision loss and/or underutilization of eye care. Based on prior literature [1, 3], we used racial/ethnic minority, residing in a rural area, low SES as social determinants for high-risk status in this study. All participants fit into one or more of these categories. Study participants were recruited from various community venues, such as predominantly Black churches, housing project communities, and free general health clinics, in Mecklenburg County, Virginia, and Durham, NC. Researchers approached individuals at these sites to request their participation in the focus group discussions. When an individual expressed interest in joining the study, they were asked their age, level of education, and date of most recent eye exam. All participants had been seen by an eye care provider in the past. Participants were classified on the basis of their most recent eye examination and socioeconomic status, using education as a proxy. The American Academy of Ophthalmology recommends that individuals 40 years old and older with risk factors for eye disease, such as Blacks, who are at higher risk for glaucoma, should be evaluated every 1 to 3 years [9]. Recent users were those who had an eye exam within 3 years, while nonusers had not had an eye exam in 3 years or more. Determining SES can be done using various determinants as proxy, such as income, wealth, education, and composite indices, in health services research [10]. Each of these methods has its own strengths and limitations [10]. Using education as a proxy is an easy, seemingly less intrusive method (compared to asking an individual's income level) and therefore chosen for this study [10]. For this study, individuals who received education beyond high school were considered to be of higher socioeconomic status, while those who did not receive postsecondary education were classified as lower socioeconomic status.

A total of 4 focus groups were conducted to provide insights into different aspects and perspectives among populations at different levels of risk for vision loss: individuals with recent eye care and lower socioeconomic status, individuals with recent eye care and higher socioeconomic status, and 2 groups with individuals without recent eye care and lower socioeconomic status. The size of the focus groups ranged from 5 to 8 people, with an average group size of 6. In all, 24 individuals participated in the groups. Of the 24 participants, 20 were women and 4 were men. There were 2 white and 22 African American participants. All of the participants lived in Durham, NC, or south central Virginia. Their ages ranged from 32 to 84 years of age, with an average age of 54 years old. Thirteen of the participants had accessed eye care within the past 3 years and 11 had not.

All of the focus groups were held at venues in the community that were easily accessible and familiar to the participants. Prior to the start of each group, informed consent was obtained from all participants. All of the focus groups were facilitated by one of the authors (A.R.E.), an African American female who was not an eye care provider (at the time of the focus groups). Her role was to pose questions to the group and facilitate the discussion. She did not have formal training in conducting focus groups. Each focus group session began with the facilitator expressing appreciation for individuals' participating, stating the objectives and “ground rules” for the focus group, and introductions by the participants. Throughout the discussion, participants were able to speak freely about their ideas on barriers to care and needed changes to the current eye care system. There were no disruptions to any of the four focus group sessions and all participants were engaged and provided comments throughout the session. All of the focus groups were recorded with 2 microcassette tape recorders for the purposes of accuracy and clarity. Participants were given a gift bag ($20 value) and eye care educational materials as a token of appreciation for their time. Each recording was later transcribed using word-processing software. Qualitative software, NUD^*^IST Vivo, was used for content analysis. In addition, the authors reviewed and analyzed each of the scripts from individual interviews and focus groups for content and key concepts. If a participant made the same comment more than once, it was only counted once.

## 3. Results

### 3.1. Barriers to Care

A total of 351 comments were made by the focus group participants in regard to barriers to receiving appropriate eye care. The most frequently cited barriers include (1) cost, (2) trust, (3) communication, (4) clinic accessibility (transportation/distance), and (5) doctor-patient relationship. These five barriers accounted for approximately 70% of all comments made about barriers to receiving eye care. In addition to the 5 most commonly cited barriers, eight other areas were identified. Examples of the comments on the most frequently cited barriers are noted in Table 1.

Cost was the most frequently cited barrier to receiving regular eye care. Combining comments on cost and insurance accounts for about 30% of all comments on barriers made in this study. Many participants mentioned that even with some form of health insurance, the cost of the exam, eyeglasses, and medications was still significant. Content analysis shows that, in the 3 groups of lower SES, cost was the most common barrier, while those of higher SES noted cost as the 6th most common.

Trust, or lack thereof, of the eye care provider and his/her motives were the next most common barrier to eye care. Many of the comments regarding lack of trust for the provider were linked with payment/cost of eye care. Some felt that they had been prescribed glasses or told they could not use their old frames to cut cost because the doctor had to “make money” and “pay the mortgage.” While trust was overall the second most common barrier, it was first among the participants who have not had recent eye care and ranked 5th among those with recent eye care.

Another important concern relating to trust was the difference in treatment based on race or SES as barriers to care. Interestingly, this may differ by age or generation. More elderly participants stated that race likely keeps some individuals from seeking eye care, while younger participants in that group disagreed with the notion. However, several participants across different ages said they were being treated poorly because of their socioeconomic status had kept them from seeing the eye doctor.

Focus group participants made it clear that poor interactions with the physician alone were not the only problem. They were also concerned with the overall service at the doctor's office, including interactions with office staff, including nurses, receptionists, and technicians. Some participants felt that some staff members treat certain patients in a disrespectful manner. In addition, long waiting times were identified as a deterrent to seeing an eye doctor.

Clinic accessibility, specifically lack of transportation and distance to eye care provider, has been identified as a significant barrier in the current and previous studies [4]. Having to travel longer distances or residing in a rural community hinders not only regular eye care use, but also appropriate care for other diseases, such as diabetes [11].

### 3.2. Suggested Changes to the Eye Care System

There were 104 comments on changes that could be made to the current eye care system made by the study participants. Suggestions on increasing educational opportunities/awareness of eye care and addressing the issues of cost and insurance were most frequently given, totaling 53% of the comments. These comments were categorized similarly to those on barriers. Examples of suggestions from each category are shown in Table 2.

Many suggested system changes were discussed with regard to education. The Department of Social Services and local churches were targeted as the most effective locations to disseminate eye care education for underutilizers. Participants also suggested “getting the word out” by having displays on marquees on local drug stores and other businesses reminding people about getting regular eye care. Other ideas included radio, newspaper, and television announcements on the importance of regular eye care, monthly mailings of educational material, and simple education by word of mouth among community members.

Ideas on eliminating cost and insurance as barriers included creating an eye care clinic for low-income patients, providing mail-in rebates after receiving an eye exam, increasing the number of free vision screenings, and having a set discount rate for patients with low incomes or some form of income adjustment. In line with the CDC [1], one participant was certain that combining regular eye examinations with other preventive healthcare practices, such as yearly general physicals, would help to decrease the barriers of cost and insurance.

Many nonusers in this study felt that eye care providers' main motivation for treating patients was financial. Having free or low-cost eye care clinics in underserved areas, as suggested by study participants, could decrease this barrier. Having eye doctors from larger institutions who go into these communities weekly or biweekly to see patients would help to build trust between the community members and providers, as long as the income and financial issues were addressed. The Lions Club was brought up as an alternative to expensive eye glasses. A related concept was the feeling that doctors needed to be “part of the community.” One participant suggested if eye doctors would hold educational workshops in the community, she/he would “feel less like a stranger” and “show more interest” in the community, leading people to seek eye care more often.

Participants discussed a need for better communication before, during, and after the visit to the doctor. For example, some mentioned going to the eye doctor for the first time and not realizing they would need someone to drive them home after receiving a dilated eye examination. Participants want doctors to explain things more clearly and in “nondoctor” terms. They suggest that providers show more concern for the patient and be more cordial. Several commented that good “bedside manner is a thing of the past” and that doctors just are not “very personable” anymore. Participants want to “be made to feel a part” of their care. Sending an informative pamphlet or having a staff member review what will take place during the initial appointment entails may alleviate some of the fear and anxiety that was identified as a barrier to care in this study. Also, receiving follow-up communication from the eye care provider in between regular visits, such as a phone call to check on patient or mailing information on their diagnosis, was suggested as a means to improve appropriate utilization.

Providing incentives, such as tuition reimbursements or signing bonuses, for eye care providers to practice in underserved communities was suggested as a means to increase the number providers in those communities. At least half of the participants who recently accessed care traveled one to two hours to be seen by an ophthalmologist at major academic institutions in Richmond, VA, or Durham, NC. For this reason, many felt that satellite eye care clinics of these institutions should be placed in surrounding underserved communities or providers from these institutions could be sent to practice in these communities once a week to decrease the barrier of distance and transportation. Participants felt strongly that if there were more eye doctors in their neighborhoods, more people would seek regular care.

## 4. Conclusions and Relevance

This pilot study sought to not only identify barriers to utilization of regular eye care in high-risk individuals, but to also learn from their perspectives what changes could be made to the eye care system to make it more likely that individuals at high risk for vision loss and underutilization will seek care. The focus group method was used for a variety of reasons. Focus groups are useful when seeking information to design a large-scale quantitative study and when seeking a range of ideas or feelings from a particular group of people [12]. The composition of a focus group is meant to be homogeneous in regard to some characteristic that is important to the researcher and not meant to be representative of the general population [12]. All of the of the participants in this study possessed one or more characteristics placing them at high risk for underutilization of care and vision loss, namely, racial/ethnic minority, lower SES, and age >65 years [3]. Those participants residing in a rural area had an additional risk factor for underutilization. In addition, focus groups were held in locations where participants would be comfortable and facilitated by an individual with whom participants can relate to on some level [12].

Cost being the most frequently cited barrier to regular eye care in this study is a finding comparable to other studies on barriers to eye care [7, 8]. As this study was done prior to the implementation of the Affordable Care Act, perhaps cost/insurance would be less of a barrier if the study were repeated today. Now that millions of individuals who had no or inadequate health insurance are insured, we hope this will significantly decrease cost and lack of adequate insurance as barriers to eye care.

Trust as a barrier to eye care has been previously documented. Owsley et al. [4] identified trust as a major barrier to eye care among a population of rural older African Americans. Honesty was found to be the most important expectation for eye care in patients at an academic eye center, further illustrating the desire for a trustworthy provider [13]. Distrust for the system is certainly not specific to eye care and has been an increasing problem in healthcare in the past several decades [14]. Participants' suggestions on increased community presence of eye care providers and alliances with organizations already trusted in the community and eye care providers would improve the level of trust felt by patients.

Trust seemed to be an underlying theme in several of the barriers and suggested changes to the eye care system elicited in this study. Of the nonusers in this study, they all (with one exception) had seen an eye care provider at some point in the past. However, they had not returned in 3 years or more, citing a variety of reasons, with trust being cited more often than cost. One example mentioned was the lack of trust due to differences in race or SES. Some even felt they were treated poorly based on these factors. As racial/ethnic minorities and individuals of lower SES are significantly less likely to receive regular eye care than whites and those of higher SES [2, 15–19], these are issues that need to be addressed.

Poor interactions with the eye care provider, including communication and patient-doctor relationship, made up about 20% of the comments on barriers to eye care. Deficits in these areas add to the distrust for the eye care system and providers felt by many underutilizers of care in this study. Lack of clear communication and a good relationship with the provider has previously been indicated as a barrier to receiving eye care or an expectation of patients [4, 8, 13].

Education was the most common suggestion on how to increase eye care utilization. One can see the importance of trust in this category as well. Local churches and the Department of Social Services are entities in the community that most people know and trust as places where the disadvantaged can seek help. While the church has been identified in previous studies as a means to increase health education [20–22], using the Department of Social Services as a focal point for eye care education is an idea that has not been explored. These suggestions demonstrate that the message source is important in facilitating trust in the message content. While participants want the information to come from eye care providers, they want it to be done in a place they feel comfortable. Previous work has shown educational interventions do not increase eye care utilization in older Black patients in Alabama after one year [23]. While eye care education is an important piece of the puzzle, increasing eye care utilization will require interventions targeting multiple barriers to care.

There are some important differences to be noted when comparing recent eye care users to nonusers (Table 3). Recent users cited trust as a barrier to care much less frequently than nonusers, suggesting that perhaps addressing this issue could result in a larger conversion of nonusers to users than with other barriers besides cost. Recent users of higher SES focused mainly on educational interventions as the most important changes to be made to the current eye care system, while nonusers of lower SES felt the majority of system changes needed were with respect to cost of eye care and insurance.

There were a number of limitations to the current study. Focus groups are not meant to be representative of the population, but hypothesis generating, eliciting new ideas for future research. As such, the current study population was not representative of the general population of underutilizers of eye care. The majority of the study participants were women. The majority of the study participants lives in rural areas and therefore may face different barriers and require different changes to the eye care system to improve their utilization than inner city populations [8, 24, 25].

This pilot study was designed to identify the suggestions of high-risk individuals on ways to improve the eye care system in an effort to improve utilization. A summary of participants' suggested changes to the current system is found in Table 4. While significant changes have been made, including the attempt to address barriers of health care cost and insurance by the Affordable Care Act, key findings in this study suggest that there is still much work to be done to address the barriers to care. Even if the cost and lack of insurance as barriers are completely eliminated, this study and others demonstrate that we still have a ways to go. Creating systems that engender trust will be a key component. A focus on educational interventions at both the community and individual levels is needed to increase awareness of the importance of regular eye care and decrease the incidence of vision loss and blindness. Increased presence of eye care providers in underserved communities may help to alleviate the barriers of distance to provider and lack of transportation. More emphasis on establishing a good doctor-patient relationship and effective communication needs to be placed in all levels of medical training. Effective communication skills should be a part of continuing education for all medical professionals and staff.

## Figures and Tables

**Table 1:** Examples of comments on most frequently identified barriers.

Barrier (% of total comments, *n* = 351)	Example
Cost (24.0%)	“It's about the finances. They wouldn't let the doctor see me because I didn't have the $20…They want all of the money before the doctor sees you, you know, so that knocks a lot of us in this community out of it.” (Focus group 1, participant 4)

Trust (15.5%)	“But I'm not gonna trust these doctors who are all about the money. They tell us we need glasses when we don't…It's not my care that they're going after that day. It's the number of patients they see that day to make their quota and be able to make the payroll. Trust is key.” (Focus group 3, participant 4)
	“All the hospitals around here, nobody should be going blind. They need to start treating people b/c they want to and b/c they care, not so they can make money. I've seen animals get better treatment than some of us poor folks, and we don't even go around biting people!” (Focus group 4, participant 5)

Communication (12.5%)	“I don't want him to be talking to me about words that I don't understand. Just tell me exactly what's going on in a language I can understand…You know, explain it very well. Don't try to go over my head and I don't understand.” (Focus group 2, participant 2)

Distance/transportation (10.2%)	“And transportation to be able to get to the eye doctor also. A lot of people don't have their own transportation, then you have to ask someone and pay for them to take you.” (Focus group 2, participant 2)

Doctor-patient relationship (8.7%)	“…they are not sincere. They don't have any feelings for you…They're not concerned about the person…I'm a whole person!” (Focus group 1, participant 6)
	“…If you go to the grocery store and the cashier is rude, you don't wanna go back to that store no more. Same thing with the doctor. Except you'll probably go back to the store b/c you gotta eat. You don't have to go to the doctor.” (Focus group 4, participant 5)

Lack of knowledge/education about eye care (6.1%)	“We hear about the heart, the lungs, kidneys, cancer, all of that, but we never hear about the eyes…we don't know what we need to do to take care of our eyes. We don't know enough about prevention in order to keep our eyes.” (Focus group 3, participant 4)

Insurance (5.8%)	“…And the first thing is “Do you have insurance?” And if you don't have insurance, you might have an appointment, but they're gonna turn you away.” (Focus group 3, participant 4)
	“Look, the only time I've ever experienced staff that's cranky and rude is when I didn't have insurance…” (Focus group 4, participant 4)

Service at the doctor's office (5.8%)	“They tell you to be there at some early time in the morning and you get there and you wait for hours! Our time is valuable too! Maybe I'm not an eye doctor but I have valuable things to do, like work to feed my family.” (Focus group 3, participant 3)
	“An unfriendly doctor or unfriendly staff will keep me from going back every time.” (Focus group 4, participant 3)

Procrastination (5.0%)	“I think we have a tendency to put it off unless they have a problem directly with seeing. But just for an eye exam, I think we have a tendency to put it off.” (Focus group 2, participant 5)

Other (2.9%)	“There are some practices that don't want new patients. So where do you start?” (Focus group 3, participant 4)
	“That's why someone who knows the person should go with them [to the eye doctor] b/c I don't feel like they would receive the same treatment as someone who knows what's going on…that's one thing that really discourages me about doctors…I don't think you should treat anybody different or put them down b/c of their economic status and things of that nature. But it happens.” (Focus group 2, participant 2)

Problems with vision devices (1.7%)	“Well I think the glasses damaged my eyes. I was not having any problems until I wore those glasses for about a week. Now I take them off and I can't see nothing.” (Focus group 3, participant 6)

Race (1.3%)	“It's not pointed out as a factor. But deep down, it's still a little bit of tension there. It's not supposed to be a factor.” (Focus group 2, participant 5)

Fear (0.7%)	“You know, I just have a fear… That's why I'm debating whether I'm going back or not…” (Focus group 3, participant 5)

**Table 2:** Examples of suggested changes to current eye care system.

Category of change (% of total comments, *n* = 104)	Example
Education (33.6%)	“Social Services Agency can be used as focal points to make educational presentations. You can do that by contacting the workers there first, so that, with the customer's permission, the social worker can present the names of people who really need the information.” (Focus group 2, participant 3)
	“You know how they have Fire Prevention Week? Is there an Eye Disease Prevention Week? Maybe we need something like that for the eye. Maybe something though the mail. Or maybe they could send something every month…Stress the importance of eye care.” (Focus group 2, participant 4)
	“I think if you start with children, there won't be a problem. By the time they're adults, they're so used to going to the eye doctor, it's nothing to them.” (Focus group 4, participant 5)

Cost/insurance (19.2%)	“…you're supposed to have a yearly physical right? Why can't they combine that? Why can't your eye exam be a part of your yearly exam?…as far as insurance or paying goes?” (Focus group 1, participant 5)
	“Well if we had a clinic, funded by the government or whoever, and we had to pay, you know $5 or $10, even $25 or $30. Just something so low-income people could afford to not go blind.” (Focus group 1, participant 1)

Distance to doctor/transportation (11.2%)	“It goes back to incentives to get doctors to come to this area…are any programs for eye doctors that give incentives, whether they're financial or tuition repayment or whatever, to work in these underserved areas for *X* number of years once you've finished? And who knows, while working there, they might get attached and want to stay. So I think the way to get more doctors here won't come from anything the communities themselves can do. It has to come from outside…there are some people…from communities like this who feel invested enough to come back and help us, but they are few and far between.” (Focus group 2, participant 3)
	“We need more doctors in our community. Or maybe some sort of satellite clinic that brings good doctors to the area. So if Duke is mass-producing good doctors, send some of them our way! Attach themselves to other facilities, put themselves in the community. So that people won't have the barrier or distance or transportation.” (Focus group 3, participant 4)
	“Well maybe we could have a volunteer service…we'll volunteer to take people to get their eyes examined. Then more people would volunteer for another month and just do it like that. Through our churches would work. The church has to take care of its people. A lot of people are retired and would love to get out and do something to help others…” (Focus group 2, participant 4)

Social support (10.2%)	“I think we're aware of preventive eye care and we just don't do what we need to do. Maybe those who are aware or familiar can get a buddy, a senior citizen that's your buddy, a person that you could look out for. If you have to take them to the eye doctor, help them keep up with their appointments, and so forth…A buddy system is my suggestion.” (Focus group 2, participant 2)

Communication (9.1%)	“I want them to ask me questions, about how I'm doing. And then about my eyes. Explain things well. And then go on and tell me what we're going to do. Answer my questions with honest answers. If they do that, we'll have no problems.” (Focus group 2, participant 4)
	“Talk to me! Sit down and talk to me. Explain what you're gonna do. The last doctor, he told me what he was gonna do, what he was gonna check…and that's exactly what he did. So I felt real comfortable with him.” (Focus group 3, participant 6)

Doctor-patient relationship (8.1%)	“…If people are made to feel like they are apart of what you're doing to them and for them, you'll get cooperation.” (Focus group 3, participant 4)
	“I think it's their attitude. They should be a little nicer and be concerned more about the patient.” (Focus group 1, participant 6)
	“…don't talk down to me. Make sure the doctor is patient and willing to explain any terms that are unfamiliar.” (Focus group 2, participant 3)

Government (5.1%)	“With all the money the government has, why not have an eye care card? Something where people could go to and access that…have a listing of programs that would serve for that plan. It would at least give people a window to get what they need for the eye.” (Focus group 3, participant 4)

Service at clinic (4.0%)	“I mean, they do need a different way to schedule patients. They schedule everyone to come in at 8 am knowing they're not gonna see them until 3 in the afternoon!…my father…he's old, sitting around for 5 or 6 hours, that's hard. So regardless of whether the eye doctor's good or not, you're already angry!” (Focus group 3, participant 3)
	“Well I'm looking at it not just from the doctor, but the entire office. When I walk in the door, do the receptionists and nurses greet me with a smile? Are they polite to me? You know, telling me what's going to happen. If the doctor is running late, you know they acknowledge it and apologize…It would be a perfect visit.” (Focus group 2, participant 3)

**Table 3:** Differences in ranking of barriers among recent and nonusers.

Recent users	Barrier	Nonusers
2nd	Communication	3rd
1st	Cost	2nd
3rd	Distance/transportation	7th
4th	Doctor-patient relationship	5th
7th	Education/knowledge	6th
8th	Insurance	4th
6th	Service in clinic	8th
5th	Trust	1st

**Table 4:** Summary of suggested changes to increase eye care utilization.

Patient-doctor interactions	
Be more courteous, patient, and understanding	
Explain things clearly and in nondoctor terms	
Follow up with patients in between visits	
Always explain the problem, treatment plan, and options; patients want to feel a part of their care	
Cost/insurance	
Create more eye care clinics for low-income people	
Make eye examinations part of yearly physical for insurance purposes	
Offer in-house financing or payment plans	
Offer mail-in rebates after eye examinations	
Offer more treatment options/programs to assist with purchasing eye glasses	
Increase number of free vision screenings in underserved communities	
Accessibility	
Develop satellite clinics of larger hospitals in underserved communities	
Have doctors from larger hospitals practice in already established facilities in underserved communities once or twice a week	
Provide incentives for eye care providers to practice in underserved communities	
Have mobile units for eye care	
Notify local board of supervisors of need for more eye care providers in community	
Volunteer transportation in community	
Education/knowledge of eye care importance	
Monthly mailings on eye care education	
Create eye disease prevention week with daily postal mailings (modeled after fire prevention week)	
Use department of social services and churches as focal points for educational interventions	
More educational seminars in the community led by providers or students	
Create community eye care awareness groups run by members of the community	
Use various forms of media (newspaper, radio, or television) to increase eye care awareness	
Use marquees on local businesses to increase awareness	
Community/social support	
Implement eye care “buddy system”	
Get family members to encourage each other about importance of eye care	
Set up community committees/organizations for eye care issues	
Service at clinic	
Find better way to schedule patients to decrease waiting time	
Train all personnel to treat patients courteously	
Government	
Make lawmakers and political candidates more aware of the problems with receiving eye care	
